# Do the same genes underlie parallel phenotypic divergence in different *Littorina saxatilis* populations?

**DOI:** 10.1111/mec.12883

**Published:** 2014-09-08

**Authors:** A M Westram, J Galindo, M Alm Rosenblad, J W Grahame, M Panova, R K Butlin

**Affiliations:** * Animal and Plant Sciences, University of SheffieldSheffield, S102TN, UK; † Departamento de Bioquímica, Genética e Inmunología, Facultad de Biología, Universidade de Vigo36310, Vigo, Spain; ‡ Chemistry and Molecular Biology, University of GothenburgSE-405 30, Gothenburg, Sweden; § School of Biology, University of LeedsLeeds, LS2 9JT, UK; ¶ Department of Biological and Environmental Sciences – Tjärnö, University of GothenburgSE-452 96, Strömstad, Sweden

**Keywords:** adaptive divergence, parallel evolution, speciation, transcriptome scan

## Abstract

Parallel patterns of adaptive divergence and speciation are cited as powerful evidence for the role of selection driving these processes. However, it is often not clear whether parallel phenotypic divergence is underlain by parallel genetic changes. Here, we asked about the genetic basis of parallel divergence in the marine snail *Littorina saxatilis*, which has repeatedly evolved coexisting ecotypes adapted to either crab predation or wave action. We sequenced the transcriptome of snails of both ecotypes from three distant geographical locations (Spain, Sweden and United Kingdom) and mapped the reads to the *L. saxatilis* reference genome. We identified genomic regions potentially under divergent selection between ecotypes within each country, using an outlier approach based on *F*_ST_ values calculated per locus. In line with previous studies indicating that gene reuse is generally common, we expected to find extensive sharing of outlier loci due to recent shared ancestry and gene flow between at least two of the locations in our study system. Contrary to our expectations, we found that most outliers were country specific, suggesting that much of the genetic basis of divergence is not shared among locations. However, we did find that more outliers were shared than expected by chance and that differentiation of shared outliers is often generated by the same SNPs. We discuss two mechanisms potentially explaining the limited amount of sharing we observed. First, a polygenic basis of divergent traits might allow for multiple distinct molecular mechanisms generating the same phenotypic patterns. Second, additional, location-specific axes of selection that we did not focus on in this study may produce distinct patterns of genetic divergence within each site.

## Introduction

Both theoretical and empirical work show that strong divergent selection may facilitate adaptive divergence and speciation even in the face of gene flow (Smadja & Butlin 2011; Weissing *et al.* 2011; Nosil 2012). Parallel divergence in distinct geographical locations characterized by similar environmental transitions is strong evidence for the role of natural selection: the repeated evolution of divergent adaptive phenotypes and reproductive isolation is unlikely to be driven by chance alone (Schluter & Nagel 1995; Johannesson 2001). Such patterns have been observed in a wide range of model systems used in speciation research, including sticklebacks (Rundle *et al.* 2000), stick insects (Nosil *et al.* 2002), periwinkles (Johannesson *et al.* 2010) and whitefish (Bernatchez *et al.* 2010).

Parallel phenotypic divergence, however, does not allow for inferences about the degree of genetic parallelism (Elmer & Meyer 2011; Faria *et al.* 2014). To what extent do repeated divergence processes rely on the same genetic variation to produce similar phenotypic outcomes?

Three major evolutionary processes may provide the genetic variation utilized by divergent selection in a given location: pre-existing standing genetic variation, gene flow from other locations and de novo mutations (Faria *et al.* 2014). With gene flow or shared standing genetic variation, divergence may have the same genetic basis in different locations. In contrast, novel mutations may generate similar divergent phenotypes, which may be produced by different mechanisms at the molecular level. Multiple processes may play a role in the same system. For example, standing genetic variation present at the *Eda* locus in marine three-spined stickleback populations is repeatedly associated with plate armour reduction during freshwater/marine divergence (Colosimo *et al.* 2005). On the other hand, different, independently evolved alleles at the *Pitx1* locus contribute to pelvic reduction during freshwater colonization in the same system (Chan *et al.* 2010). Overall, the limited data currently available, across taxa, suggest that reuse of the same loci is common (Conte *et al.* 2012).

Studying the relative contribution of genetic parallelism and nonparallelism will improve our understanding of speciation and the factors that facilitate it (Elmer & Meyer 2011). Theory predicts multiple factors that may have an influence: effective population sizes, demographic history (e.g. bottlenecks) and the history of selection and gene flow may affect the amount of standing genetic variation available; geographical conditions affect the extent of gene flow between locations; and mutation rates and effective population sizes influence the availability of novel adaptive mutations (Hartl & Clark 1997). Additionally, genetic parallelism might be more pronounced in recently diverged taxa, which are expected to share more standing genetic variation. Furthermore, genetic constraints may limit the range of possible evolutionary pathways, and these constraints may be more similar in closely related taxa (Conte *et al.* 2012).

Recent advances in high-throughput sequencing technology have the potential to contribute greatly to understand the extent of genetic parallelism (Elmer & Meyer 2011). In the early stages of adaptive divergence with gene flow, genomic regions affected by divergent selection are expected to show higher differentiation compared with the rest of the genome, in which allele frequencies can be homogenized by gene flow. This difference is utilized in genome scans to identify ‘outliers’, genomic regions potentially affected by divergent selection (Wilding *et al.* 2001; Luikart *et al.* 2003; Beaumont 2005). By comparing sets of outlier loci obtained from different geographical locations, it is possible to estimate the extent to which the same genomic regions are under divergent selection (Hohenlohe *et al.* 2010; Deagle *et al.* 2012; Kautt *et al.* 2012).

Recent studies using such approaches have shown that the same genomic regions are often involved in divergence in multiple locations (Bonin *et al.* 2006; Nosil *et al.* 2008; Hohenlohe *et al.* 2010; Jones *et al.* 2012). These patterns may be due to the reuse of shared standing genetic variation (Seehausen *et al.* 2008; Roberts *et al.* 2009; Jones *et al.* 2012; Bradic *et al.* 2013). For example, in the stickleback marine–freshwater divergence, the same alleles are often reused across large geographical scales (Jones *et al.* 2012). The prominent role of standing genetic variation in rapid adaptation may be explained by the fact that it provides an immediate target for selection to act on, while adaptation based on new mutations might take much longer (Barrett & Schluter 2008).

There has also been evidence for the role of gene flow in parallel phenotypic divergence. Genetic variation pre-existing or emerging by mutation in one location may spread to other locations, especially if a selective advantage eases introgression (Morjan & Rieseberg 2004; Hedrick 2013). In some cases, such alleles may fuel local divergence, as found in *Heliconius* butterflies (Heliconius Genome Consortium 2012).

On the other hand, there are cases where phenotypic parallelism is not generated by the same molecular mechanism (i.e. few or no outliers are shared among locations), even if divergence is relatively recent (Renaut *et al.* 2011; Deagle *et al.* 2012; Kautt *et al.* 2012; Roda *et al.* 2013; Soria-Carrasco *et al.* 2014).

**Figure 1:**
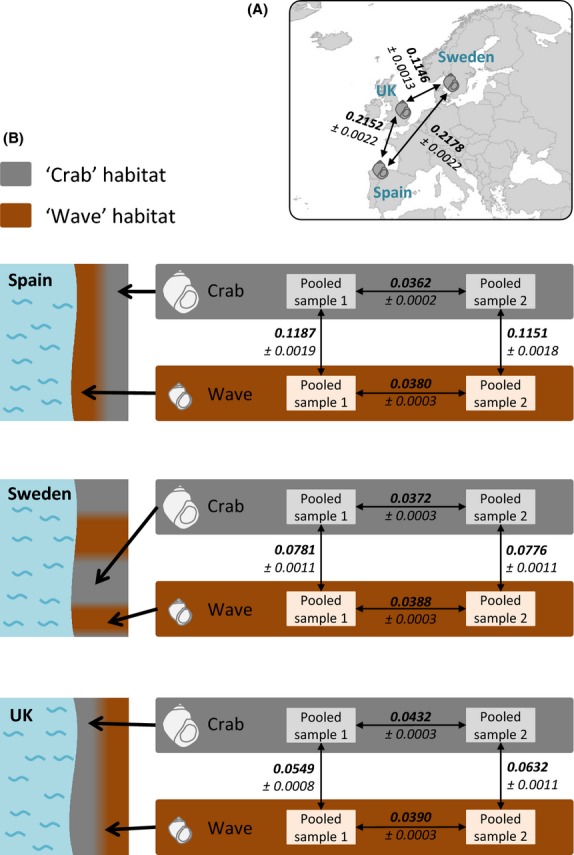
(A) Geographical location of the three sites sampled in this study and estimates of *F*_ST_ between them (average ± SE across all loci; *n* = 6790). (B) Left: Schematic shore topology at the three sampled locations (grey: ‘crab’ habitat, brown: ‘wave’ habitat). Right: *F*_ST_ estimates between replicate pooled samples from the same ecotype and between pooled samples from different ecotypes (average ± SE across all loci; *n* = 6790).

Here, we study parallel divergence in the marine snail *Littorina saxatilis*, a model system for divergent adaptation and speciation. *Littorina saxatilis* inhabits shores across Europe and North America. It occurs in a variety of habitats, including rocky areas such as boulder fields and steep cliffs, but also in salt marshes and soft substrates in brackish water (Reid 1996). Most studies so far have focussed on two rocky shore ecotypes, adapted either to crab predation or to wave action (Rolán-Alvarez 2007; Butlin *et al.* 2008; Johannesson *et al.* 2010), which occur on distinct parts of the same shores. Such ecotype pairs can be found in geographically distant locations on shores with quite distinct topologies (Fig. 1B). We will refer to these ecotypes as ‘crab ecotype’ and ‘wave ecotype’, even though additional selection pressures (which may vary among locations) may also play a role in their divergence. They differ phenotypically from each other along multiple axes, including shell characteristics (e.g. shell thickness, aperture size), behaviour (wary vs. bold) and size (crab ecotype larger) (Johannesson *et al.* 2010; Butlin *et al.* 2014). Hybridization occurs in relatively narrow contact zones (few metres), but gene flow is limited due to assortative mating, immigrant inviability and habitat choice (Johannesson *et al.* 1995b, 2010; Rolán-Alvarez 2007; Webster *et al.* 2012).

At neutral markers, genotypes cluster by geographical location instead of by ecotype, even within countries (Johannesson *et al.* 1993; Rolán-Alvarez *et al.* 2004; e.g. Panova *et al.* 2006; Quesada *et al.* 2007; Galindo *et al.* 2009, 2013), and a recent study using approximate Bayesian computation and a large data set of neutral markers suggests that in situ divergence is likely, even when locations within the same country are considered (Butlin *et al.* 2014). Still, it is not clear whether the loci targeted by divergent selection are the same across sites and whether locally adaptive alleles share a common origin.

No study so far has looked at the extent of adaptive genetic parallelism in *Littorina* on large geographical scales. Here, we sequenced the *L. saxatilis* transcriptome, including snails from three geographical locations: two populations probably sharing a recent postglacial origin (Sweden and United Kingdom) and a southern European one that is more genetically distinct (Spain) (Doellman *et al.* 2011; Panova *et al.* 2011; Butlin *et al.* 2014). We identified outliers between ecotypes for each location and asked how many of these are shared among the different geographical locations.

## Methods

### Sampling

*Littorina saxatilis* was collected in three locations (Fig. 1A), in Spain (Silleiro; 42.1012°, −8.8972°), Sweden (Tjärnö; 58.8650°, 11.1305°) and the United Kingdom (Thornwick Bay; 54.1328°, −0.1129°), between December 2011 and January 2012. Snails from the ‘wave’ and the ‘crab’ habitats were sampled separately, avoiding contact zones. Within habitats, similarly sized adult individuals were sampled haphazardly along a ∼25-m stretch of shore. Snails were dissected and only females carrying embryos in their brood pouch were used further. Only these females can be clearly distinguished from a cooccurring (in the UK), morphologically cryptic sister species (*L. arcana*), which lays eggs (Reid 1996).

### RNA extraction, pooling, sequencing and bioinformatics

We sequenced pools of RNA from multiple individuals per location and ecotype. With this approach, it is possible to include a larger number of individuals for the same cost to get a more accurate representation of population allele frequencies, at the expense of individual genotype information (Futschik & Schlötterer 2010; Zhu *et al.* 2012). RNA extraction and pooling are described in Appendix S1 (Supporting information). In total, we prepared twelve different pools (three countries × two ecotypes × two replicates), each containing RNA from ∼40 female snails and their embryos (typically tens to hundreds per female; Janson 1985). These pools should cover a wide range of genes due to the presence of different developmental stages (even though male-specific genes may be under-represented).

Barcoded RNAseq libraries for the twelve pools were prepared using Illumina TruSeq RNA Sample Prep Kit v2 using 1 μg of total RNA input and 10 PCR cycles, as per the manufacturer's recommendations. They were sequenced in a single lane, using an Illumina HiSeq 2000 machine (100 bp paired-end reads; insert size around 285 bp), at Edinburgh Genomics, Edinburgh, UK. Raw reads were filtered as described in Appendix S1 (Supporting information). A total of 148.7 m were retained (between 7.5 and 18.9 m reads per sample).

A draft reference genome of *L. saxatilis* is available (from one ‘crab’ ecotype snail from Sweden; The IMAGO Marine Genome project, http://www.cemeb.science.gu.se/research/imago-marine-genome-projects/; project coordinated by Anders Blomberg and Kerstin Johannesson). We mapped the RNAseq reads to this reference genome using the mapper gsnap (Wu & Nacu 2010), allowing for the identification of novel splice sites (novelsplicing = 1). Potential overlap between paired-end reads was clipped (clip-overlap). Mapping was run four times, allowing for different maximum proportions of mismatches per read (4%, 6%, 8% or 10% of each read).

We only kept reads that mapped to a single location in the genome and to the same reference contig as their paired-end read (‘concordant_uniq’ files output by gsnap). Using these criteria, for a maximum of 8% mismatches, between 60% and 68% of the reads for each sample were retained (between 4.6 and 12.0 m reads per sample; total 96.7 m reads). There was no pronounced difference between countries with regard to the average proportion of reads in a sample that could be mapped (Spain: 64%, Sweden: 66% and UK: 65%).

Mapping files were sorted and converted to the BAM format using samtools (Li *et al.* 2009). samtools mpileup was used to generate an mpileup file, which contains allele counts for each base position, using only reads with a mapping quality of at least 20. This file was converted to the ‘sync’ format associated with the popoolation2 package (Kofler *et al.* 2011), discarding bases with a base quality lower than 20 and removing positions containing deletions.

### *F*_ST_ calculation

We analysed the data at the level of the reference contig to limit the pseudoreplication associated with treating SNPs as independent units. Because the *L. saxatilis* genome is in a draft state, the contigs are relatively short (N50 = 950 bp; average contig length 660 bp) and unlikely to contain more than one gene, probably representing one or a few exons. Differentiation between ecotypes should be increased not only at SNPs targeted directly by selection, but also at closely linked SNPs, because selection locally reduces effective gene flow between ecotypes (Charlesworth *et al.* 1997); therefore, integrating information about differentiation along the whole contig is a way of obtaining more reliable candidate loci.

Because RNAseq data sets are characterized by a large variation in coverage depth across loci, the data were subsampled to an even coverage of 20 per sample (i.e. per pool of 40 individuals + embryos) using the subsampling with replacement strategy in popoolation2 (Kofler *et al.* 2011; details in Appendix S1, Supporting information). SNPs were then identified, applying a minor allele count threshold (across all 12 samples; i.e. both shared and population-specific SNPs may be included in the data set) to remove sequencing errors and uninformative SNPs (Roesti *et al.* 2012). Expected heterozygosity within and between samples was averaged over all retained SNPs within a contig, and used to calculate *F*_ST_ for that contig (see Appendix S1, Supporting information for further details).

As we had two ‘crab’ and two ‘wave’ pools per location, we obtained two estimates of *F*_ST_ between ecotypes (crab 1–wave 1 and crab 2–wave 2) for each country. We also calculated *F*_ST_ within ecotypes (crab 1–crab 2 and wave 1–wave 2), as well as *F*_ST_ between countries. We are aware that popoolation2 is not intended for *F*_ST_ calculation from transcriptome data, where individual contribution to the pools may vary due to expression differences; however, our samples contained large numbers of individuals, and the replicates produced generally similar results (see below), supporting the validity of our approach.

### Outlier identification

For the contigs that passed filters, our first aim was to identify those with exceptionally high *F*_ST_ between ecotypes within each country. The distinction between ‘outliers’ and ‘nonoutliers’ is arbitrary, and the models underlying classical genome scan methods may be violated by demographic history and the ubiquity of purifying selection in the transcriptome causing variation in effective mutation rate (Charlesworth 1998). We therefore simply used a quantile of the *F*_ST_ distribution for a given sample pair as a threshold for outlier identification, as has been done in some previous studies (e.g. Renaut *et al.* 2012). Within each country, contigs were only considered outliers when both replicate *F*_ST_ estimates were above the threshold quantile to obtain a more robust set of outliers. As the true fraction of loci influenced by divergent selection was unknown, we repeated the analysis with several different threshold quantiles (between 94% and 98%).

Outliers shared between countries (‘shared outliers’) were those contigs that were identified as outliers in both focal countries for a given threshold. Some extent of sharing would be expected even if outliers were drawn randomly from the total set of contigs. Therefore, we also determined the expected number of shared loci if sharing was due to chance only, as well as its 95% confidence limits, using a hypergeometric distribution function (Paterson 2002; Roda *et al.* 2013). A fact that has not received much attention is that gene flow within ecotypes between locations may generate a (weak) correlation of allele frequencies, so that estimates of *F*_ST_ between ecotypes might rank more similarly in different locations than expected by chance. In theory, this could generate shared ‘outliers’ even without the effect of selection. We have analysed this effect in more detail and conclude that it is not likely to have a major impact on our results (Appendix S2, Supporting information).

All outlier identification analyses were conducted using custom r (R Core Team 2013) and Python scripts.

### SNPs within outlier contigs

*F*_ST_ values calculated per contig do not reveal whether differentiation in shared outliers is due to differentiation at the same nucleotide position. To analyse this, we asked whether allele frequency differences between ecotypes at SNPs in shared outlier contigs were correlated between countries (see Appendix S1, Supporting information). If the same SNP alleles are associated with the ‘crab’ ecotype in both countries, there should be a positive correlation. If SNP alleles are associated with the ‘crab’ ecotype in one, but the ‘wave’ ecotype in the other country, there should be a negative correlation. If different SNPs are associated with the divergence in the two countries, the correlation should on average be zero. Note, however, that positive and negative correlations will emerge at random if different SNPs are involved in the differentiation; therefore, it is only meaningful to study overall patterns instead of results for individual contigs. Significance was tested by bootstrap, analysing whether the mean Pearson's correlation coefficient of outlier contigs differed from that of all contigs (Appendix S1, Supporting information). We also grouped loci into bins based on their correlation between countries and performed a chi-square test asking whether the distribution across bins was different from that observed for all loci (Appendix S1, Supporting information).

## Results

### Differentiation between ecotypes and countries

The number of reference genome contigs included in *F*_ST_ calculations and downstream analyses varied according to the stringency of settings during bioinformatic analyses (minimum: 4584, maximum: 9649). In addition, mean *F*_ST_ values increased with increasing minor allele count used for SNP identification, probably because SNPs with very small minor allele counts can necessarily only produce small *F*_ST_ values (Roesti *et al.* 2012). However, patterns of outlier sharing (which we focus on here) were largely unaffected. We therefore only report results obtained when using a mapping threshold of 8% mismatches and a minor allele count of 24 (10%) over all samples for SNP identification (6790 contigs). The relatively large mismatch rate we used is appropriate in *L. saxatilils*, which is known to be characterized by high rates of genetic polymorphism (Butlin *et al.* 2014).

**Figure 2:**
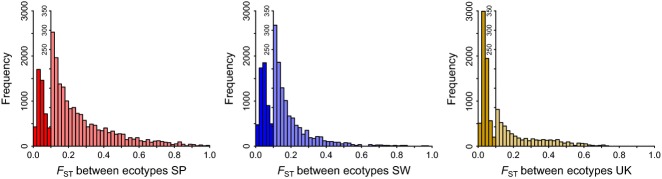
*F*_ST_ estimates between ecotypes for all loci within each of the three countries (SP: Spain, SW: Sweden, UK: United Kingdom). A second *y*-axis with a larger scale is introduced for *F*_ST_ values >0.1 to emphasize the tail of the distribution. *n* = 6790 loci.

When all contigs were considered, average *F*_ST_ between samples from the same country and ecotype was lower than between-ecotype *F*_ST_, as expected (Fig. 1B). Spain showed the highest level of differentiation between ecotypes (Figs 1B and 2). For all three countries, the long tail of the *F*_ST_ distribution reflects loci that might be affected by divergent selection (Fig. 2).

*F*_ST_ between countries was higher on average than between ecotypes within a country, with differentiation between Sweden and the UK being lower than between Spain and either of the two other countries (Fig. 1A).

**Figure 3:**
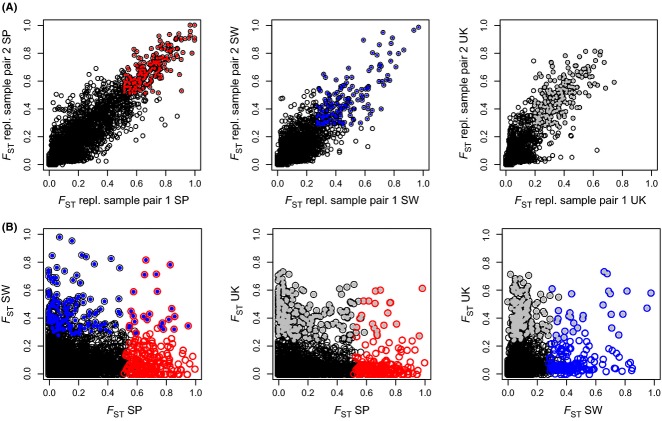
Correlation of between-ecotype *F*_ST_ estimates between replicate sample pairs and between countries. (A) For each locus, we obtained two *F*_ST_ estimates per country (*F*_ST_ of crab–wave sample pair 1 and *F*_ST_ of crab–wave sample pair 2). The plots show the relationship between these two estimates within each country (left: Spain, middle: Sweden, right: UK). (B) Relationship of between-ecotype *F*_ST_ estimates between countries (*F*_ST_ averaged across the two replicate estimates within countries). Codes are as in Fig. 2. Loci above the 96% quantile of the *F*_ST_ distribution in both replicate sample pairs (‘outliers’) are shown in colour (Spain: red, Sweden: blue, UK: grey). *n* = 6790 loci.

*F*_ST_ estimates were strongly correlated between replicate sample pairs, that is crab–wave sample pairs from the same country (Pearson's correlation coefficients: Spain: 0.91, Sweden: 0.83, UK: 0.86, Fig. 3A). In contrast, correlations of between-ecotype *F*_ST_ estimates between countries were much less pronounced (Pearson correlation coefficients: Spain–Sweden: 0.18, Spain–UK: 0.14, Sweden–UK: 0.25, Fig. 3B).

### Sharing of outliers

**Figure 4:**
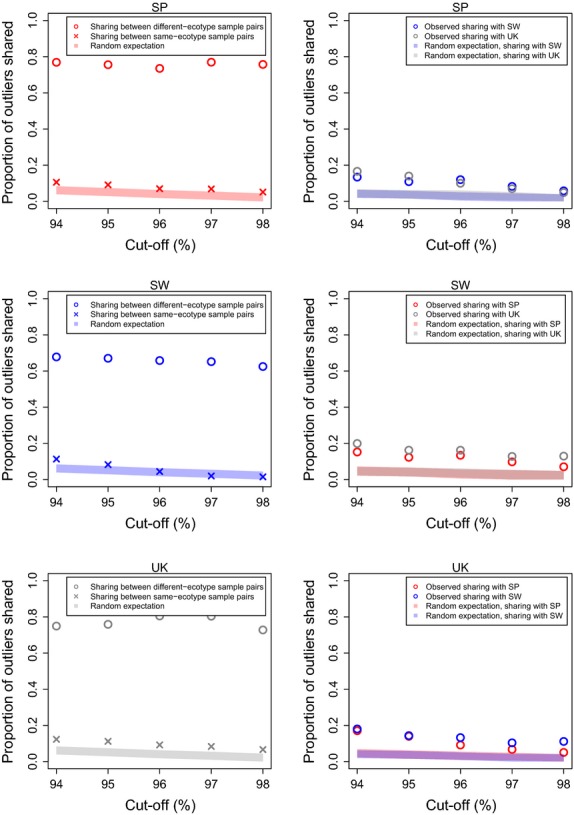
Proportions of outliers that were shared between sample pairs within countries, or between the focal country and the two other countries, at different thresholds used for outlier identification (focal country: top: Spain, middle: Sweden, bottom: UK). Left side: Sharing between sample pairs within countries. Circles: Sharing between replicate sample pairs (i.e. between crab–wave sample pair 1 and crab–wave sample pair 2); crosses: sharing between nonreplicate sample pairs (i.e. between a crab–crab sample pair and a wave–wave sample pair). Right side: observed sharing between two countries (circles). Only outliers that are shared between replicate sample pairs within each country are included. Shaded area (both sides): 95% CI of the proportion of shared outliers expected by chance. *n* = 6790 loci.

For each outlier detection threshold, we counted contigs shared between replicate sample pairs. In this case, any lack of sharing should be due to experimental noise (including sampling of individuals contributing to the pools, among-individual variation in the amount of extracted RNA and variation in gene expression). For quantiles between 94% and 98%, more than 60% of outliers were consistently shared between replicate sample pairs, while the expectation for sharing due to chance is substantially lower (<10%) and decreases with increasing quantiles (Fig. 4, left side).

On the other hand, pairs of samples from the same ecotype within a country (i.e. sample pairs in which no real outliers should be observable) should only have shared ‘outliers’ by chance. As shown in Fig. 4 (left side), sharing in this case was typically below 10%, and within or slightly above the range expected.

One of our main goals was to estimate the extent to which outliers were shared between countries. Although the *F*_ST_ quantile above which a contig was considered an ‘outlier’ was arbitrary, we focused on contigs with *F*_ST_ above the 94% quantile, based on visual inspection of the plots (Fig. 4) and previous studies showing that typically between 5% and 10% of markers were outliers (reviewed in Nosil *et al.* 2009).

The number of outliers shared between countries was always larger than the random expectation: about 4× more outliers were shared than expected by chance. For example, for the 94% quantile, shared outliers represented a fraction of 13–20% of the total number of outliers found within a country (Fig. 4B, right side; also see Fig. 3B), while only about 4% of the contigs would be expected to be shared by chance.

If only the contigs with *F*_ST_ above the 98% quantile were considered, the proportion of outliers shared with another country decreased to 5–13%, but was still larger than expected by chance (Fig. 4B, right side). This decrease is expected because even if outliers are shared among countries, they do not necessarily rank similarly regarding their extent of differentiation. Therefore, with increasingly stringent cut-offs, different loci will be excluded, reducing the observed extent of sharing.

Notably, as Fig. 4 shows, the extent of sharing was similar for all three possible sample pairs. Despite their more recent shared history and lower general differentiation, Sweden and the UK did not share considerably more outliers with each other than with Spain.

Sharing among all three locations was limited to a very small number of loci (94% cut-off: six loci, 96%: three loci, 98%: zero loci).

### SNPs within outlier contigs

**Figure 5:**
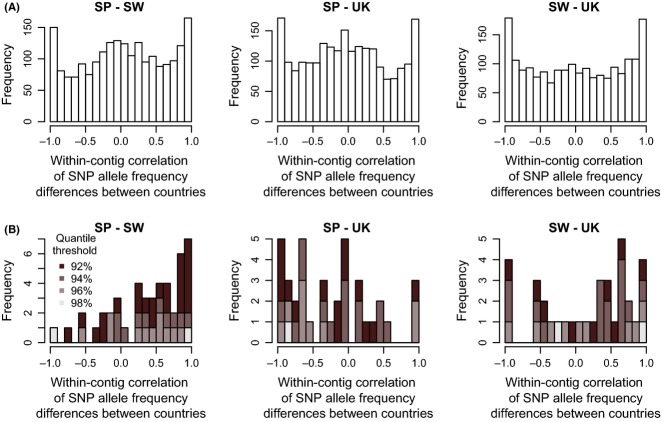
Within-contig correlations of SNP allele frequency differences between countries, for all loci (A) and only for outliers shared by two focal countries at the 92% (black), 94%, 96% and 98% (pale grey) quantile thresholds (B). Only contigs with more than two SNPs were included (Spain–Sweden: *n* = 2146, Spain–UK: *n* = 2256, Sweden–UK: *n* = 1956).

The average correlation of allele frequency differences per contig between countries was close to zero if all contigs were considered (Fig. 5A). This is in accordance with expectations for neutral loci, in which no association between genotypes and ecotypes is expected. Strong positive or negative correlations could be observed (Fig. 5A), but were similarly common (the relatively large number of correlations near −1 and 1 is expected by chance if there are many contigs with only three SNPs).

However, shared outlier contigs showed a pattern deviating from the rest of the contigs. For the Spain–Sweden and the Sweden–UK comparison, positive correlations were more common than negative ones (Fig. 5B), indicating that the same SNPs tend to be differentiated in the same direction between ecotypes in both countries. However, this was significant only for the Spain–Sweden comparison at the lower thresholds (bootstrap test: 92% and 94%; chi-square test: 92%, *P* < 0.01). In contrast, in the Spain–UK comparison, most contigs showed a negative correlation (bootstrap test: significant at 92% and 96%; chi-square test: 92%: *P* < 0.05; 96%: *P* < 0.05), suggesting that the same SNPs have diverged in different directions.

## Discussion

The genomic basis of divergent adaptation and speciation is currently a topic under much debate (Seehausen *et al.* 2014). The extent to which parallel divergence on the phenotypic level is explained by genetic parallelism has been studied in relatively few systems (e.g. Colosimo *et al.* 2005; Seehausen *et al.* 2008; Chan *et al.* 2010; Conte *et al.* 2012; Gagnaire *et al.* 2013; Roda *et al.* 2013). Nevertheless, available data suggest that reuse of genes may be common, especially where repeated divergence occurs in closely related populations or species (Conte *et al.* 2012). Genome scans comparing pairs of populations under divergent selection, including an early AFLP-based study of *Littorina saxatilis* in the UK (Wilding *et al.* 2001), have often found many outlier loci to be shared between comparisons (Nosil *et al.* 2009). Therefore, we expected extensive reuse of genes between regions, especially between Swedish and UK populations. Contrary to our expectations, our results reveal very few shared outliers: more than expected by chance, but only by a small margin, and similar proportions of shared outliers in all three comparisons.

Our methodology may have failed to reveal the true extent of gene reuse during ecotype formation. We chose a pooling approach for transcriptome sequencing to include a larger number of individuals. Uncertainty in allele frequency, and hence *F*_ST_ estimates may have reduced our ability to detect shared outliers. However, uncertainty should be reduced if pools are large, because individual differences in contribution cancel each other out (Futschik & Schlötterer 2010). In RNAseq data, however, the contribution from each individual cannot be controlled because of variation in gene expression levels. The subsampling approach we applied partly accounts for this. More importantly, we used two replicate sample pairs per location. Reassuringly, *F*_ST_ estimates were strongly correlated between replicates, and most outliers were identified in both (Figs 3A and 4), indicating that our approach is reliable.

A minor drawback of analysing sequence variation in transcriptome data is that some loci may show allele-specific differences in gene expression (Knight 2004). If these differences are ecotype or habitat dependent, such loci cannot be distinguished from loci with true allele frequency differences. However, these are still loci of interest in the sense that they effectively generate a difference between ecotypes that may be due to *cis*-acting substitutions in many cases. On the other hand, there are some outlier loci we might have missed, given that our data represent only a portion of the transcriptome. At present, we cannot estimate what fraction of all expressed genes is included in this study; however, the total number of loci (∼7000) should ensure that a wide variety of functions is covered. There may be a bias towards higher expression levels, as low-expression transcripts may often be missed in RNAseq unless sequencing coverage is very deep. These low-expression transcripts might include genes that contribute to divergence through differential expression (the subject of a separate ongoing study; M. Panova, T. Johansson, B. Canbäck, A. Sa-Pinto, K. Johannesson & C. André, unpublished). While we included various tissues from embryos as well as adult individuals, genes predominantly expressed in adult males might be absent and could be interesting for future studies. Our filtering may also have excluded genes from rapidly expanding gene families. Reads associated with such genes may have been mapped to multiple paralogues, especially given that we had to allow for a large number of mismatches (because of the high diversity in our study species), leading to their exclusion from the data set.

We found genetic differentiation between countries and ecotypes consistent with expectations from previous studies (Wilding *et al.* 2001; Panova *et al.* 2006; Galindo *et al.* 2009, 2010, 2013; Butlin *et al.* 2014), further demonstrating the reliability of the data. Differentiation between Sweden and the UK was lower compared with their differentiation from Spain, probably reflecting the more recent shared colonization history of the northern European locations (Panova *et al.* 2011). Differentiation between ecotypes within countries was lower than differentiation between countries, consistent with recent evidence for in situ emergence of the ecotypes (Butlin *et al.* 2014). Genetic differentiation between ecotypes was highest in Spain and lowest in the UK. It is possible that the Spanish ecotypes had more time to accumulate differences as their current habitat could be colonized earlier than the northern locations, which were ice-covered during the last glacial maximum (Clark *et al.* 2012; Butlin *et al.* 2014).

Detection of loci influenced by divergent selection using genome-scan approaches is subject to both false negatives and false positives (Excoffier *et al.* 2009; Hermisson 2009; Butlin 2010). One reason for false positives is that factors other than divergent selection, such as mutation rate and recombination rate, vary across the genome and may affect *F*_ST_ estimates (Nachman & Payseur 2012). For example, large genomic regions may exhibit low heterozygosity due to a combination of low recombination rate and selection (positive or negative), causing outliers via low genetic diversity within rather than large divergence between populations (Cruickshank & Hahn 2014). Because such factors may be conserved features of the genome, they might influence *F*_ST_ estimates similarly across locations leading to sharing of ‘outliers’. False positives may also result from features of the population history (e.g. population structure) that are not captured by the outlier method used (Excoffier *et al.* 2009), and population history also influences the rate of false negatives. Also, loci with different evolutionary histories may differ with regard to their detectability in genome scans. For example, recent sweeps (induced by new mutations) within ecotypes leave more pronounced signatures at linked sites, and therefore are more likely to be detected, than old polymorphisms. To guard against these effects, we considered a range of outlier detection thresholds. If truly selected loci are shared, they should represent a higher proportion of outliers detected at high stringency. As we saw little change in the extent of outlier sharing with detection threshold (Fig. 4), we consider these artefacts unlikely to account for the low level of reuse of genes.

We suggest two explanations that could contribute to the surprising pattern we observe: polygenic inheritance and multiple dimensions of selection. If divergent traits are polygenic (such as size and shell shape in *Littorina*), there may be multiple different pathways towards similar phenotypic outcomes. Even though adaptation utilizing standing genetic variation may be expected (Barrett & Schluter 2008), the large effective population sizes of *L. saxatilis* may mean that segregating variation is present at many loci, with different subsets involved in adaptation in different regions (observed as ‘cycling’ of loci in simulations; Yeaman & Whitlock 2011). Local extinction and recolonization, which has been observed in *Littorina* following algal blooms (Johannesson & Johannesson 1995), may cause temporary local changes in the available genetic variation, accentuating differences in the outcome of selection. A large effective population size may increase the supply of new mutations and the effectiveness of selection, leading to divergence at different loci. Most current evidence for reuse of genes is based on alleles of large effect because the majority of studies are based on QTL or candidate-gene approaches (Conte *et al.* 2012), so a lower level of reuse in polygenic traits may be common.

There is good evidence for a similar contrast between the habitats occupied by crab and wave ecotypes of *L. saxatilis* in the three regions in terms of predation and wave exposure (reviewed in Johannesson *et al.* 2010). However, other dimensions of selection may differ between regions: for example, the crab ecotype occupies the higher tidal level in Spain, but the lower tidal level in the UK, and there is almost no tide in Sweden. There is also phenotypic evidence for location-specific patterns of divergence; for example, the shells of Spanish ‘crab’ snails are ridged, while those of ‘wave’ snails are smooth. Such differences in shell sculpture are absent in Sweden and the UK (Johannesson *et al.* 2010). For these reasons, outlier contigs might not necessarily be affected by the parallel selection pressures we focus on, but by other sources of divergent selection that differ among countries (i.e. nonparallel divergence, Kaeuffer *et al.* 2012; also see below), thus reducing the expected extent of sharing. Outliers may also correspond to intrinsic incompatibilities ‘trapped’ at environmental boundaries (Bierne *et al.* 2011), which are independent of the environment and may therefore not be shared across regions.

Little sharing of outliers has also been found in other systems of recent parallel phenotypic divergence (e.g. Renaut *et al.* 2011; Deagle *et al.* 2012; Kautt *et al.* 2012). These systems have similarities and differences with the *Littorina* system. All focus on cases where there is divergence in multiple, continuously variable phenotypic traits that are likely to have a polygenic basis. However, the crater-lake cichlids (Kautt *et al.* 2012) experienced bottlenecks during colonization that are likely to have resulted in different samples of genetic variation, further reducing expected reuse of loci. Conversely, a shared allopatric phase in the history of whitefish populations (Renaut *et al.* 2012) might be expected to have increased genetic parallelism. There is potential for different axes of selection in different regions in all cases.

When outlier identification is performed on the SNP level, it is possible that shared targets of selection are missed because directly selected SNPs show different associations with marker alleles among the populations compared. As we analysed mean *F*_ST_ across contigs, this is unlikely to have contributed to the low level of sharing of outliers that we observe. However, where we do see shared outlier contigs, it is possible that the underlying differentiation at the SNP level is not the same between regions: that is, different, perhaps independently evolved alleles at the same loci may contribute to parallel divergence (as in the case of *Pitx1*, Chan *et al.* 2010). We tested this possibility by examining the correlations between levels of differentiation for individual SNPs within shared outliers. We did find that, for the Spain–Sweden and Sweden–UK comparisons, many of the shared outliers showed a positive correlation of between-ecotype SNP allele frequency differences (Fig. 5B). This indicates that the same allele is favoured in the same ecotype in both countries. The SNP patterns we found for the Spain–Sweden and Sweden–UK comparison may therefore indicate a shared evolutionary history at least for some of the loci under divergent selection, as in other systems (Colosimo *et al.* 2005; Seehausen *et al.* 2008; Jones *et al.* 2012). These correlations also provide evidence that the loci involved are not false positives.

However, the pattern for the Spain–UK comparison was different: here, SNP correlations for outlier contigs were often negative, indicating that alleles common in the Spanish ‘crab’ ecotype are common in the ‘wave’ ecotype in the UK, and vice versa (Fig. 5B). One possible explanation is that, as mentioned above, some of the outliers may respond to axes of selection other than the crab–wave axis. One such axis is the high shore–low shore selection axis associated with exposure time (and therefore selection on desiccation resistance, etc.). Allozyme studies have detected divergent selection along this axis in Sweden and the UK (Johannesson & Johannesson 1989; Johannesson *et al.* 1995a; Hull *et al.* 1999). The crab–wave axis has a reversed orientation relative to the high shore–low shore axis in Spain compared with the UK. For outliers responding to high shore–low shore selection, one would therefore expect to find the alleles associated with the crab ecotype in the UK to be favoured in the wave ecotype in Spain, and vice versa.

Using the transcriptome is an efficient ‘complexity-reduction’ method to study a manageable but still widely representative set of loci and to focus on those that are expressed (Galindo *et al.* 2010; Renaut *et al.* 2013). However, patterns in the transcriptome might not necessarily be representative of the whole genome. Control regions may well be under selection and play an important role in adaptation and speciation (Wray 2007; Jones *et al.* 2012). Parallel changes in gene expression have been observed, for example in whitefish (Derome *et al.* 2006; St-Cyr *et al.* 2008).

While we focus on general patterns of parallelism here, our analysis has revealed many loci potentially under selection, some in the same direction in more than one region, some in opposing directions and some in only one location. It will be interesting to follow up individual outlier loci in more detail. A limitation for our study is that the *L. saxatilis* reference genome, to which our RNAseq reads were mapped, is currently in a draft state and consists of short, unannotated contigs. Annotation is complicated by the limited amount of genomic information available from related molluscs. Future work will allow for the functional classification of outlier loci, which will contribute greatly to our understanding of the molecular mechanisms underlying parallel divergence in these snails. For example, even if different genomic regions are involved (as shown in this study), these might still be associated with the same functional pathways (Roda *et al.* 2013).

With a more advanced genome assembly, it will also be possible to understand better the genomic architecture of outlier loci; and further studies, for example using mapping approaches in natural hybrid populations (Lindtke *et al.* 2012; Malek *et al.* 2012), will show to what extent outlier loci are associated with divergent phenotypic traits.

## Data accessibility

Raw RNAseq reads are available on Dryad, doi:10.5061/dryad.21pf0. The 6790 genome contigs used in the final analysis will be made available on Dryad with an embargo of 1 year (as the *L. saxatilis* genome is currently unpublished). Mpileup files reflecting the mapped reads will be included as well.

## Supporting Information

Appendix S1 Additional information about materials and methods.

Appendix S2 Effect of gene flow on the neutral expectation for shared outliers.
