# Supplementary material for: Do the same genes underlie parallel phenotypic divergence in different Littorina saxatilis populations?
Source: Mol Ecol. 2014 Sep 8;23(18):4603–16. doi: 10.1111/mec.12883 (PMC4285301; doi:10.1111/mec.12883)
Supplement: Appendix S1 — Additional information about materials and methods. [file mec0023-4603-SD1.docx]

Do the same genes underlie parallel phenotypic divergence in different *Littorina saxatilis* populations?

Authors: Westram AM, Galindo J, Alm Rosenblad M, Grahame JW, Panova M, Butlin RK

Supplementary Information S1

## RNA extraction and pooling

Prior to extraction, shells were removed and individual female snails (including their embryos) were placed in 1.5 ml microcentrifuge tubes containing 1ml Trizol reagent (Invitrogen) and a 5mm steel bead. Tubes were shaken in a tissue lyser for 2x5min at 25Hz, and then centrifuged at 13,000rpm for 10min. Supernatants (containing the RNA) of between 13 and 18 female snails of similar size were pooled in equal amounts to obtain a total pool volume of 1ml. RNA was then extracted following the manufacturer's instructions. Afterwards, RNA concentration was measured using an Agilent 2100 BioAnalyzer, and two or three of the initial pools were combined in equal concentrations, so that we ended up with two replicate pools per site and ecotype. Each of the twelve final pools contained RNA from between 32 and 48 female snails and their embryos. The pools were DNase treated (TURBO DNA-free DNase kit) and purified, applying the Qiagen RNeasy Mini Kit according to the manufacturer’s instructions.

## Read quality filtering

Data were quality trimmed using the programs sickle and scythe (https://github.com/najoshi/sickle, https://github.com/vsbuffalo/scythe), using a quality cut-off of 20 and retaining sequences longer than 50bp, then split by barcode. Sequences containing Ns were discarded.

## F_ST_ calculation

Because RNAseq datasets are characterised by a large variation in coverage depth across loci, allele counts were randomly subsampled to obtain an even coverage depth of 20 per base position and sample (subsampling with replacement in PoPoolation2 ; Kofler *et al.* 2011). Base positions with an initial coverage depth lower than 20 were discarded. Subsampling was repeated 50 times. Within each of the 50 subsample datasets, SNPs were identified as variable positions where the minor allele count (across all 12 samples) was larger than a set threshold (4, 24, or 48 - i.e. 1.67%, 10%, or 20% of the total coverage depth).

In PoPoolation2, F_ST_ calculation is SNP-based, discarding information about potential linkage disequilibria. Expected heterozygosity per pooled sample (H_s_) and expected heterozygosity across pooled samples (H_t_) are first calculated per SNP. Then H_s_ and H_t_ are each averaged across the SNPs in a contig. These average values are used to calculate F_ST_ = (H_t_-H_s_)/H_t_ (Hartl & Clark 1997). For all contigs that contained at least one SNP (on average across all subsample data sets), per-contig F_ST_ was calculated this way within each subsample data set, and then averaged across subsample data sets.

## SNPs within outlier contigs

We first calculated average allele counts for each SNP in each sample, using the 50 subsample data sets mentioned above. For each biallelic SNP, we then chose an arbitrary allele and calculated the allele frequency difference between ecotypes (frequency in crab ecotype – frequency in wave ecotype) within each country. This difference was averaged over the two replicate sample pairs (i.e. the two crab-wave sample pairs from the same location), so that we obtained one value per country and SNP.

As in the outlier identification analysis, we applied a minor allele count to exclude uninformative SNPs. Because only the focal population pair (as opposed to all three countries) was considered in each SNP-wise analysis, the total number of alleles was 160 (2 countries x 2 ecotypes x 2 replicate pooled samples x coverage depth of 20). We excluded SNPs with a minor allele count < 16 in the focal population pair (i.e. 16/160 alleles; equivalent to the intermediate threshold of 10% used for outlier identification).

Within each contig that contained more than 2 SNPs in the focal comparison, we calculated the Pearson correlation of allele frequency differences between two countries. Significance of the average correlation for outlier loci was tested by randomly drawing the same number of loci from the complete set and calculating the average correlation for each of the randomly sampled data sets. This process was repeated 1000 times to generate a distribution of expected average correlations. Observed values below the 2.5% quantile, or above the 97.5% quantile of this distribution were considered statistically significant. Additionally, we performed chi-square tests comparing the distribution of outliers across correlation bins of size 0.4 (i.e. 5 bins in total, between -1 and 1) to the distribution of all loci. This test focuses on the distribution of outliers, rather than the mean correlation, and should therefore be more sensitive to situations where e.g. both strongly negative and positive correlations are more common in outlier loci, while the mean is not necessarily shifted. Bin sizes of 0.4 were chosen in order to obtain a reasonable number of loci per bin. Tests were performed in R (R Core Team 2013).

**References**

Hartl D, Clark A (1997) *Principles of polulation genetics*. Sinauer Associates, Inc.

Kofler R, Pandey RV, Schlötterer C (2011) PoPoolation2: identifying differentiation between populations using sequencing of pooled DNA samples (Pool-Seq). *Bioinformatics*, **27**, 3435–3436.

R Core Team (2013) R: A language and environment for statistical computing.
