# Supplementary material for: Do the same genes underlie parallel phenotypic divergence in different Littorina saxatilis populations?
Source: Mol Ecol. 2014 Sep 8;23(18):4603–16. doi: 10.1111/mec.12883 (PMC4285301; doi:10.1111/mec.12883)
Supplement: Appendix S2 — Effect of gene flow on the neutral expectation for shared outliers. [file mec0023-4603-SD2.docx]

Do the same genes underlie parallel phenotypic divergence in different *Littorina saxatilis* populations?

Authors: Westram AM, Galindo J, Alm Rosenblad M, Grahame JW, Panova M, Butlin RK

Supplementary Information S2

## Effect of gene flow on the neutral expectation for shared outliers

Given that samples have been taken from each of two ecotypes, here denoted W (‘wave’) and C (‘crab’), from each of two regions, here 1 and 2, we consider the proportion of F_ST_ outliers that would be expected to be shared between the pairs W1-C1 and W2-C2 under neutrality. Outlier status is based on a percentile cut-off, *c*. If the regions are demographically independent, then the expected proportion of shared outliers (i.e. outlier loci observed in both comparisons as a fraction of the number of outliers in the comparison that has fewer outliers) is simply (1-*c*/100), which is used as the ‘neutral expectation’ in the main text. However, any lack of independence due to gene flow between regions or a common ancestral population will result in a correlation between allele frequencies in different regions and so in F_ST_ estimates. This may, in turn, cause the proportion of shared outliers to exceed the expectation under independence. Previous studies reporting shared outliers have not considered this possibility.

To test the magnitude of this effect in data comparable to those analysed here, we used the coalescent simulator fastsimcoal2.1 (Excoffier & Foll 2011). We simulated samples of *n* alleles from each of 4 populations of constant size 10 000 diploid individuals: W1, C1, W2, C2. Symmetrical migration occurred between pairs W1-C1 and W2-C2 at rate *m*_WC_ and between pairs W1-W2 and C1-C2 at rate *m*_12_. No migration was allowed between W1 and C2 or between C1 and W2. In one set of simulations (‘with migration’), we set *m*_12_ > 0 and the demographic scenario was stable back to the most recent common ancestor (MRCA). In the other set (‘no migration’), we set *m*_12_ = 0 and all four populations were derived from a single ancestral population of size 40 000 at time *t*. In each case, we simulated 10 000 loci, each of length 1000 bp, mutation rate 5x10^-9^ and unbiased transition-transversion ratio. For simplicity, free recombination was allowed between loci and no recombination within loci. F_ST_ was estimated for each locus in all pairs of populations using Arlequin 3.5.1.3 (Excoffier & Lischer 2010). We included in subsequent analyses only loci that were polymorphic in both regions such that both between-ecotype F_ST_ estimates were non-zero.

The simulations conducted are summarised in Table S1 and the impact on sharing of outliers is summarised in Figure S1. The proportion of shared outliers only fell outside the confidence interval for the expected proportion when gene flow between regions was high (*Nm* = 1 giving F_ST_ between regions ~0.068). Even this effect was lost at high percentile cut-offs for outliers or when sample size was low. Shared ancestry, even as recent as 2000 generations before sampling, did not generate an excess of shared outliers.

We conclude that gene flow between regions can generate sharing of false positive outliers, i.e. those neutral loci at the extreme of the F_ST_ distribution, but that this effect is not likely to contribute to sharing in our study because gene flow between regions is too low (F_ST_ > 0.1).

Table S2. Simulation parameters and realised average F_ST_ values.

| **Simulation** | ***n*** | ***m*_WC_ x10^5^** | ***m*_12_ x10^5^** | ***t* (generations)** | **Loci analysed** | **Mean F_ST_ across loci** | | | |
| --- | --- | --- | --- | --- | --- | --- | --- | --- | --- |
|  |  |  |  |  |  | **W1-C1** | **W2-C2** | **W1-W2** | **C1-C2** |
| Sim_15_10 | 20 | 15 | 10 | - | 8398 | 0.0554 | 0.0541 | 0.0679 | 0.0665 |
| Sim_15_05 | 20 | 15 | 5 | - | 8244 | 0.0590 | 0.0582 | 0.110 | 0.109 |
| Sim_15_02 | 20 | 15 | 2 | - | 7943 | 0.0620 | 0.0631 | 0.204 | 0.203 |
| Sim_15_10_n50 | 50 | 15 | 10 | - | 9082 | 0.0548 | 0.0543 | 0.0692 | 0.0686 |
| Sim_15_02_n50 | 50 | 15 | 2 | - | 8865 | 0.0639 | 0.0620 | 0.209 | 0.209 |
|  |  |  |  |  |  |  |  |  |  |
| Sim_NoMig_10000 | 20 | 15 | 0 | 10 000 | 7328 | 0.0666 | 0.0676 | 0.182 | 0.184 |
| Sim_NoMig_2000 | 20 | 15 | 0 | 2 000 | 8207 | 0.0485 | 0.0498 | 0.0628 | 0.0627 |
| Sim_NoMig_2000 | 50 | 15 | 0 | 2 000 | 9042 | 0.0506 | 0.0498 | 0.0667 | 0.0648 |


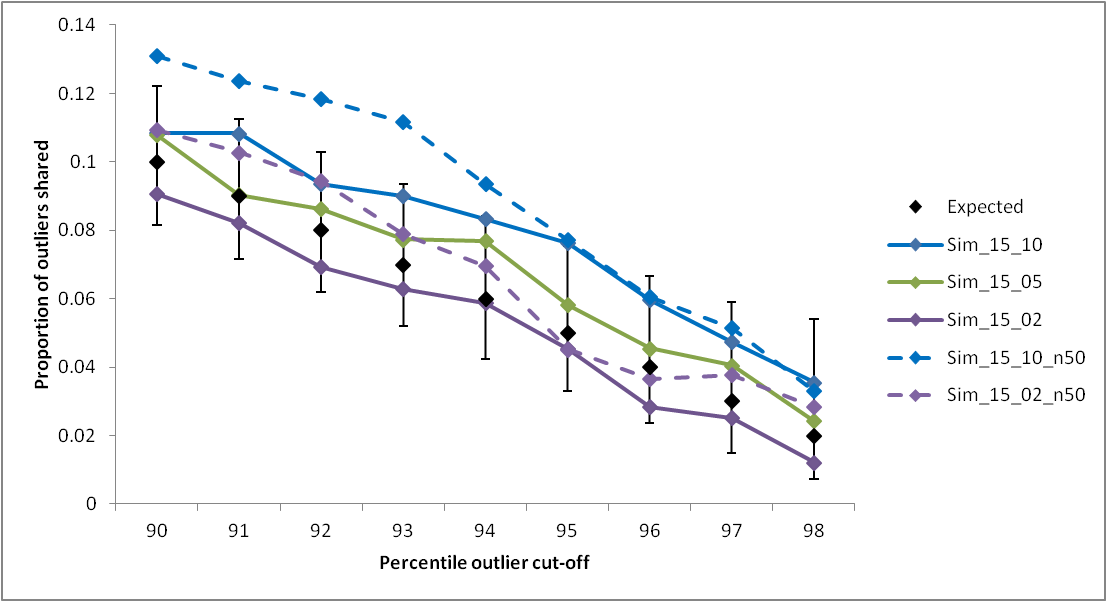


Fig. S2A

Results of simulations with migration between sites: Proportion of outliers shared among two locations for different stringencies of outlier detection. Simulation parameters are indicated in the legend and explained in Table S1.


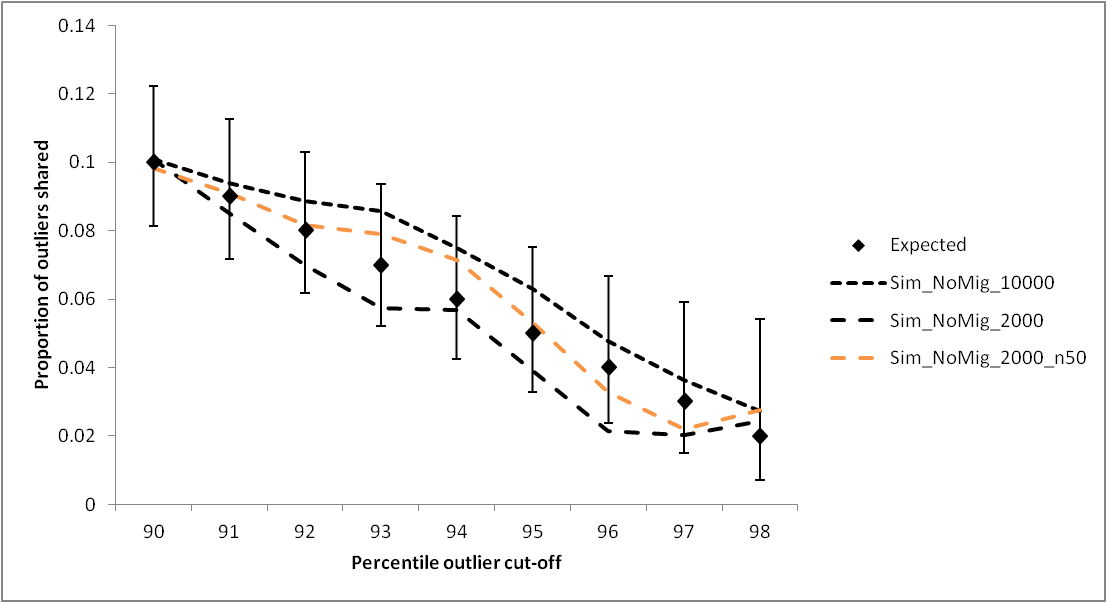


Fig. S2B

Results of simulations without migration between sites: Proportion of outliers shared among two locations for different stringencies of outlier detection. Simulation parameters are indicated in the legend and explained in Table S1.

**References**

Excoffier L, Foll M (2011) fastsimcoal: a continuous-time coalescent simulator of genomic diversity under arbitrarily complex evolutionary scenarios. *Bioinformatics*, **27**, 1332–1334.

Excoffier L, Lischer HEL (2010) Arlequin suite ver 3.5: a new series of programs to perform population genetics analyses under Linux and Windows. *Molecular Ecology Resources*, **10**, 564–567.
